# Clinical efficacy of retrograde urethrography-assisted urethral catheterization after failed conventional urethral catheterization

**DOI:** 10.1186/s12894-021-00788-6

**Published:** 2021-02-04

**Authors:** Si Hyun Kim, Hee Jo Yang, Doo Sang Kim, Chang Ho Lee, Youn Soo Jeon, Ki Hong Kim

**Affiliations:** Department of Urology, Soonchunhyang University Cheonan Hospital, Soonchunhyang University College of Medicine, 31 Suncheonhyang 6-gil, Dongnam-gu, Cheonan, 31151 Korea

**Keywords:** Retrograde urethrography, Urethral catheterization, Urethral injury

## Abstract

**Background:**

Several approaches for urethral catheterization after the failure of initial urethral catheterization have been introduced. However, standard procedures regarding what should be done after failed conventional urethral catheterization have been not established. Therefore, we investigated the clinical efficacy of retrograde urethrography (RGU)-assisted urethral catheterization after failed conventional urethral catheterization.

**Methods:**

Between July 2015 and July 2018, 136 patients who underwent RGU-assisted urethral catheterization after failed conventional urethral catheterization were included in this retrospective study. Patients’ clinical data, such as age, catheterization site, and previous history of urologic operations, were collected and assessed via chart review. Univariate and multivariate logistic regression analyses were performed to identify predictive factors for the failure of this procedure.

**Results:**

Of the 136 patients, 94 (69.1%) experienced successful RGU-assisted urethral catheterization. Having a previous history of urologic operations, such as urethrotomy and transurethral prostatectomy, was identified as an independent predictive factor for the failure of RGU-assisted urethral catheterization (odds ratio = 9.453, 95% confidence interval = 2.703–33.063, *p* < 0.001).

**Conclusions:**

RGU-assisted urethral catheterization can be one of the modalities for providing successful catheterization after failed conventional urethral catheterization. We believe that RGU-assisted urethral catheterization can be an effective procedure if patients have no previous history of urologic operations, such as urethrotomy and transurethral prostatectomy. *Trial registration* Soonchunhyang university institutional review board approval (No. 2018-08-021).

## Background

Urethral catheterization is one of the most common urologic procedures. For various reasons, this procedure is performed on about a quarter of inpatients [1]. However, clinicians are sometimes faced with unexpected difficulties during urethral catheterization (DDUCs). Although DDUCs are not common, if this is not properly handled, they could cause critical problems, such as urethral injury [2]. Complications associated with urethral injury are significant hazards of urinary catheterization. This causes patients to unnecessarily increase hospitalization and pay extra-medical expenses [3]. A recent study has reported that urethral catheter-related injuries occur in 1.4% of urethral catheterization patients [4], and the cost of managing these injuries was about $371,790 [5].

Further, there are many factors that can cause failed catheterization [6]. Common causes of DDUCs for normal urethras include tight external sphincters caused by anxiety in patients and poor catheterization technique. Additionally, pathologic causes include urethral stricture, phimosis, edema, bladder neck contracture, prostate cancer, false passages, and benign prostatic hypertrophy, among others [7]. Among them, most urethral injuries, particularly false passages, occur during initial catheterization. This is because external sphincter contraction occurs when the catheter enters the bulbous membranous urethra. Injuries are sometimes caused by the balloon when the catheter is not fully inserted or the catheter tip itself causes incorrect passage [4]. In these cases, the retrial of catheterization with conventional blind technique could further damage the urethra [8].

Several approaches for urethral catheterization after the failure of initial urethral catheterization have been introduced [7]. However, standard procedures regarding what should be done after failed conventional urethral catheterization have been not established. Therefore, we investigated the clinical efficacy of urethral catheterization under retrograde urethrography (RGU) guidance after failed conventional urethral catheterization and attempted to identify factors for the success of this procedure.

## Methods

### Study design

A total of 136 consecutive male patients who underwent urethral catheterization under RGU guidance between July 2015 and July 2018 were included in this retrospective study. All patients underwent RGU-assisted urethral catheterization after failed conventional urethral catheterization. After institutional review board approval (No. 2018-08-021) was granted for this study, we conducted a retrospective chart review of the included patients. All patients received written informed consent and agreed prior to the procedure. All procedures in this study were performed in accordance with the ethical standards of the institutional and national research committee and with the 1964 Helsinki declaration.

### RGU-assisted urethral catheterization procedure

**Fig. 1:**
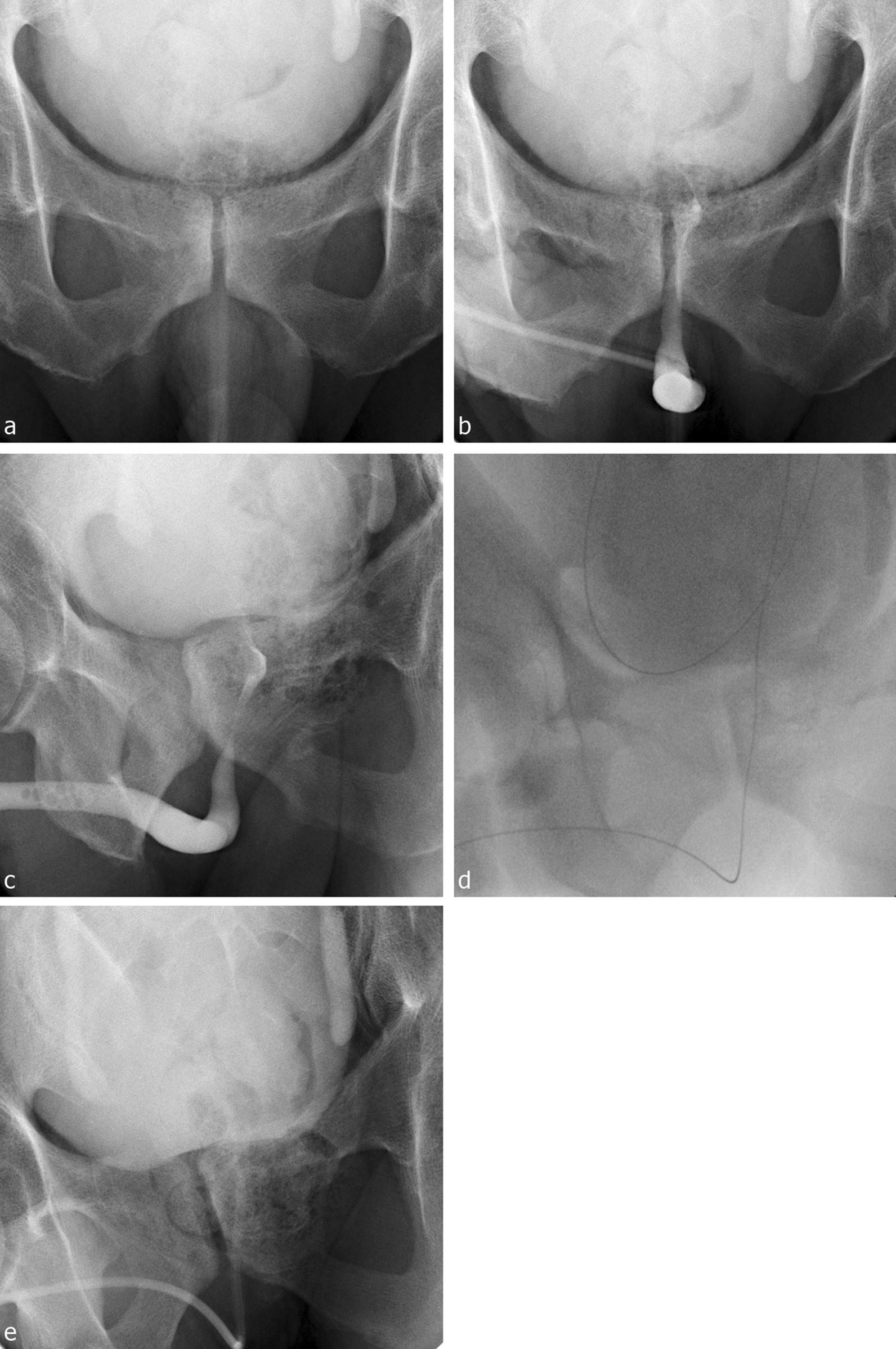
Urethral catheterization under RGU. **a** Contrast fluid remained in the bladder after enhanced computed tomography. **b** Narrowed prostatic urethra. **c** An oblique image of a narrowed prostatic urethra. **c** A hydrophilic guide wire was inserted into bladder under fluoroscopic guidance. **d** Foley catheter was inserted into the bladder using a hydrophilic guide wire

RGU using a fluoroscope was performed during urethral catheterization. Urethral catheterization was performed using a 18 French sized three-way Foley catheter with a hole that was used for continuous irrigation. Prior to the procedure, the operator wore protective equipment against radiation exposure. To start the procedure, a fluoroscope was placed in front of patients. Then, a hydrophilic guide wire (Terumo Radiofocus® Guide wire Angled, 0.35″, 150 cm) was inserted into bladder through the hole of the catheter under fluoroscopic guidance. Next, a 60-mL enema syringe was filled with 1:2 mixture of contrast solution and normal. Under RGU guidance, the syringe was then connected at the drainage hole of the Foley catheter and pressure was applied to the urethra. Finally, catheterization was completed through this guide wire (Fig. 1).

### Patients’ clinical data and statistical analysis

The following clinical data were assessed: age, past history, previous history of urologic operations, medication for lower urinary tract symptoms, site of catheterization, reason for catheterization, history of trauma, and RGU finding. Baseline characteristics were compared via ANOVA for continuous variables and via chi-square test for categorical variables. Univariate and multivariate logistic regression analyses were performed to identify predictive factors for the failure of RGU-assisted urethral catheterization. The two-sided p-values of < 0.2 and < 0.005 were considered statistically significant for the univariate and multivariate analyses, respectively. All statistical analyses were performed using SPSS Statistics version 20.0.0 (IBM Corp., Armonk, NY, USA).

## Results

In total, 136 patients underwent RGU-assisted urethral catheterization. Of these 136, 94 (69.1%) experienced successful RGU-assisted urethral catheterization and 42 (30.9%) experienced failed RGU-assisted urethral catheterization. Moreover, 18 (13.2%) patients required suprapubic percutaneous catheterization or cystoscopy-assisted urethral catheterization after failed RGU-assisted urethral catheterization, and 15 (11.0%) were treated via urethral sound dilatation after successful RGU-assisted urethral catheterization.

Patients’ baseline clinical characteristics are presented in Table 1. In our study, 18 (13.2%) patients had a previous history of urologic operations, including transurethral prostatectomy, radical prostatectomy, and endoscopic urethrotomy. Most events happened in the emergency room, and most of urethral catheterizations were performed for checking urine output, absolute bed rest or operation. Definite urethral injury under RGU was identified in 33 (24.3%) patients.

**Table 1:** Patients characteristics

Age	76.0 (65.0–81.0)
Past history	
Cerebral vascular disease	29
Heart disease	28
Pulmonary disease	47
Diabetes	20
Hypertension	50
Chronic renal failure	10
Previous urologic procedure	18
Medication for LUTS	56
Site	
ER	89
Ward	47
Reason for catheterization	
AUR	23
Routine catheterization	113
Trauma	
Perineal	122
None	11
Others	3
RGU finding	
Except urethral rupture	103
Urethral rupture	33

The complete results of our univariate and multivariate analyses are presented in Table 2. These analyses identified a previous history of urologic operations as an independent predictor of failed RGU-assisted urethral catheterization (odds ratio = 9.453, 95% confidence interval = 2.703–33.063, *p* < 0.001).

**Table 2:** Predictive factors for the failure of urethral catheterization under RGU

Univariate analysis		
Variables	OR	*p* value
Age		
Continuous	0.985 (0.957–1.014)	0.311
Categorized	1.198 (0.578–2.481)	0.627
Past history		
Cerebral vascular disease	1.175 (0.488–2.830)	0.718
Heart disease	1.024 (0.416–2.518)	0.959
Pulmonary disease	0.603 (0.271–1.340)	0.214
Diabetes	0.657 (0.221–1.953)	0.450
Hypertension	1.187 (0.554–2.545)	0.659
Chronic renal failure	0.219 (0.027–1.794)	**0.157**
Previous urologic procedure	8.200 (2.671–25.177)	** < 0.001**
Medication for LUTS	0.746 (0.349–1.597)	0.451
Site (ER vs GW)	0.674 (0.306–1.485)	0.328
Reason for catheterization (AUR vs routine)	0.514 (0.205–1.289)	**0.156**
Perineal trauma (vs none)	0.472 (0.098–2.287)	0.351
RGU finding (others vs rupture)	0.640 (0.261–1.568)	0.329

Multivariate analysis		
Variables	OR	*p* value
Chronic renal failure	0.211 (0.022–2.021)	0.177
Previous urologic procedure	9.453 (2.703–33.063)	** < 0.001**
Reason for catheterization (AUR vs routine)	0.805 (0.279–3.320)	0.688

## Discussion

Since the Seldinger technique, which involves using a guide wire to obtain safe access to blood vessels, was introduced [9], it has been applied as a second-line method after failed initial urethral catheterization [2, 10–17]. The Seldinger technique safely to indwell urethral catheter can be classified according to whether cystoscopy is used or not.

Urethral catheterization using only hydrophilic guide wires for DDUCs has been introduced [11–17]. The insertion of a hydrophilic guide wire, which helps to lead the urethral catheter to the correct location, can be performed without causing trauma to the urethra and with minimal pain to the patient [13]. Unfortunately, this technique is often unsuccessful making a false passage or tissue damage, because the location of guide wire could not be affirmed [11].

Recently, a novel Foley catheter that integrates a hydrophilic guide wire has also been introduced [2]. Physicians have advocated for the use of this device by reporting that it is relatively simple to use and its use results in high success rates with a 0% incidence rate of adverse events for nurse-led male urethral catheterization. However, it is difficult to guarantee a 100% success rate for the general population because this novel catheter has not been used in cases where slight resistance during urethral catheterization was present.

There have also been several reports about performing urethral catheterization via the Seldinger technique with a flexible cystoscope [10, 18]. The authors of such a report stated that the success rate of this method was 96% [10]. Further, Villanueva et al. reported on the common approaches that are performed when initial urethral catheterization fails [19]. Based on an online survey, urologic residents most commonly resorted to flexible cystoscopy as opposed to the blind placement of guide wires or filiforms/followers after trying one or more urethral catheters. However, Villanueva et al. mentioned that urethral catheterization with flexible cystoscopy assistance is limited in terms of economics and time saving. Further, it is important to consider the learning curve for performing flexible cystoscopy. MacKenzie et al. reported that acceptable flexible cystoscopy was achieved by the 122nd procedure [20]. In other words, this procedure can only be performed with a certain level of expertise.

Other second-line urethral catheterization techniques have also been reported aside from the Seldinger technique. One such technique involves using pressure to open the urethra [21]. For this technique, a syringe filled with normal saline or sterile water is attached to behind of a typical catheter, drainage channel. By pressing the plunger of the syringe, the hydrostatic effect of the fluid helps to separate the prostatic lobes, thereby providing the tip of the catheter easier passage into the bladder [21].

Moreover, Kameda et al. attempted using trans-abdominal ultrasonography as a second-line urethral catheterization method [22]. They evaluated whether trans-abdominal ultrasonography could show the tip of a urethral catheter and whether trans-abdominal ultrasound-guided catheterization with transrectal pressure could result in successful catheterization for male patients in whom performing standard catheterization was difficult. However, this method is difficult to perform without an experienced physician.

Minagawa et al. reported that trans-rectal ultrasonography can be useful for DDUCs [6]. It could provide more clear findings regarding the anatomy of the male urethra. However, this procedure can only be performed by experienced physicians who understand trans-rectal ultrasound findings.

In a study including 10 patients, Athanasopoulos et al. suggested that using a ureteral access sheath for urethral dilation was helpful for the catheterization of difficult urethral strictures [23]. They mentioned that the use of both a hydrophilic guide wire and ureteral sheath proved their technique’s efficacy and resulted in the atraumatic characteristics observed within the ureteral lumen. However, while successful catheterization was achieved in all patients, only a small number of patients were enrolled.

RGU-assisted urethral catheterization is a method that combined the following techniques: the Seldinger technique, fluoroscopy, and the application of hydrostatic pressure to the urethra. Therefore, the greatest advantage of this method is that it allows clinicians to clearly identify the anatomy of the urethra and evaluate the position of the guide wire. Moreover, the catheter tip could be easily inserted into the bladder because the application of hydrostatic pressure of the mixture of contrast solution and normal saline during RGU, to the urethra helped to separate the prostatic lobes. In summary, RGU-assisted urethral catheterization is a method that provides a combination of the advantages of previously reported methods. This procedure is relatively simple to implement, whereas urethral catheterization through flexible cystoscopy requires an experienced urologist.

Previous urological procedures were identified as an independent predictor for the failure of this method in multivariate analysis. Prostate transurethral resection, visual internal urethral resection, prostate holmium laser resection, and simple or radical prostatectomy are common surgeries in this category. The authors contend that these procedures resulted in an anatomical change in the urethra that the Foley catheter or guide wire could not pass through. If the patient has no history mentioned above, this method is worth considering clinically.

Our study is limited because it is retrospective in nature, it assessed only single-center data, and it did not include a large number of patients. Therefore, the results of our study must be confirmed and validated with a prospective large-scale multi-center study. Further, radiation exposure was not evaluated in our study, and a successful catheterization rate of 69.1% may not be satisfactory. However, high success rates can be expected after appropriate patient selection, as a history of previous urologic operations was identified as an independent predictor of failed RGU-assisted urethral catheterization.

## Conclusions

RGU-assisted urethral catheterization can be considered one of the methods for providing successful catheterization after failed conventional urethral catheterization. In particular, if there is no previous history of urologic operations, such as urethrotomy and transurethral prostatectomy, RGU-assisted urethral catheterization can be an effective procedure for DDUCs.

## Data Availability

The datasets used and/or analysed during the current study available from the corresponding author on reasonable request.

## Abbreviations

RGU: Retrograde urethrography
DDUC: Difficult during urethral catheterization
BPH: Benign prostatic hypertrophy
LUTS: Lower urinary tract symptoms
ER: Emergency room
AUR: Acute urinary retention
